# The Effect of Glycosaminoglycans (GAGs) on Amyloid Aggregation and Toxicity

**DOI:** 10.3390/molecules20022510

**Published:** 2015-02-02

**Authors:** Clara Iannuzzi, Gaetano Irace, Ivana Sirangelo

**Affiliations:** Dipartimento di Biochimica, Biofisica e Patologia Generale, Seconda Università di Napoli, Via L. De Crecchio 7, Napoli 80138, Italy; E-Mails: clara.iannuzzi@unina2.it (C.I.); gaetano.irace@unina2.it (G.I.)

**Keywords:** glycosaminoglycans, amyloid aggregation, amyloid toxicity inhibition

## Abstract

Amyloidosis is a protein folding disorder in which normally soluble proteins are deposited extracellularly as insoluble fibrils, impairing tissue structure and function. Charged polyelectrolytes such as glycosaminoglycans (GAGs) are frequently found associated with the proteinaceous deposits in tissues of patients affected by amyloid diseases. Experimental evidence indicate that they can play an active role in favoring amyloid fibril formation and stabilization. Binding of GAGs to amyloid fibrils occurs mainly through electrostatic interactions involving the negative polyelectrolyte charges and positively charged side chains residues of aggregating protein. Similarly to catalyst for reactions, GAGs favor aggregation, nucleation and amyloid fibril formation functioning as a structural templates for the self-assembly of highly cytotoxic oligomeric precursors, rich in β-sheets, into harmless amyloid fibrils. Moreover, the GAGs amyloid promoting activity can be facilitated through specific interactions via consensus binding sites between amyloid polypeptide and GAGs molecules. We review the effect of GAGs on amyloid deposition as well as proteins not strictly related to diseases. In addition, we consider the potential of the GAGs therapy in amyloidosis.

## 1. Introduction

Proteinaceous deposits in the tissues of patients affected by amyloid diseases have been frequently found associated with charged poly-electrolytes and other factors [1,2,3,4,5,6]. Indeed, a careful examination of the diseased tissues has revealed the presence, in the deposits, of a significant amount of polysaccharides belonging to the glycosaminoglycan family (GAGs). Among these species, heparan sulfate (HS) is the most common, being found in a variety of amyloid disorders including Alzheimer’s disease, type II diabetes, light chain amyloidosis, and prion related diseases [6,7]. The deposition of amyloid in mammals is strongly associated with several extracellular matrix components, including glycoproteins and GAGs. The distribution of these components in mammals produces different biological environments, which may affect the extent and the tissue distribution of amyloid deposition *in vivo*. Moreover, there is strong evidence that GAGs play an active role in favoring amyloid fibril formation and stabilization [8,9]. Basic concepts on the biochemistry and biology of GAGs and their implications in neurodegeneration have been recently reviewed by Papy-Garcia *et al.* 2011 [10]. Although different hypotheses have been proposed to explain the mechanisms by which GAGs could facilitate amyloid fibril formation, only little information is available, and the precise mechanism by which GAGs accelerate amyloidogenesis is still subject to debate. It has been hypothesized that, as for catalyzed reactions, GAGs favor aggregation, nucleation and amyloid fibril formation by a mechanism substantially different from that occurring in bulk solution [11]. The available data suggest that they can both influence and promote misfolding of polypeptides into pro-amyloidogenic intermediates rich in β-sheets, and also act as a structural template for self-assembly. The scaffold may function by enhancing the structural features that favor a β-sheet conformation thereby increasing the number of nucleation seeds. In the later stages of the amyloid pathway, GAGs could also enhance lateral aggregation of small fibrils conferring insolubility and protection from proteolysis [5,12]. Recent studies have shown that HS induces changes in the aggregation process by splitting it in a parallel manifold faster pathway [13].

These observations suggest that GAGs could play an active role in the amyloidogenesis *in vivo*, perhaps even a protective role, by conversion of proteotoxic soluble oligomers into less toxic amyloid fibrils and related cross-β-sheet aggregates. In this review, we analyze some of the most recent results showing that proteins containing exposed clusters of basic residues may undergo amyloid-like aggregation in the presence of GAGs. This feature is also shared by proteins not associated to amyloid diseases.

## 2. Amyloid Aggregation

Amyloid diseases are related to anomalies in the folding process of proteins that form insoluble fibril deposits. Indeed, the aberrant assembly of one of more than 40 human proteins into insoluble fibrillar deposits is the hallmark of human amyloid diseases, among which there are both neurodegenerative disorders such as Alzheimer’s disease, and non-neuropathic conditions such as type-II diabetes [14,15,16].

**Figure 1:**
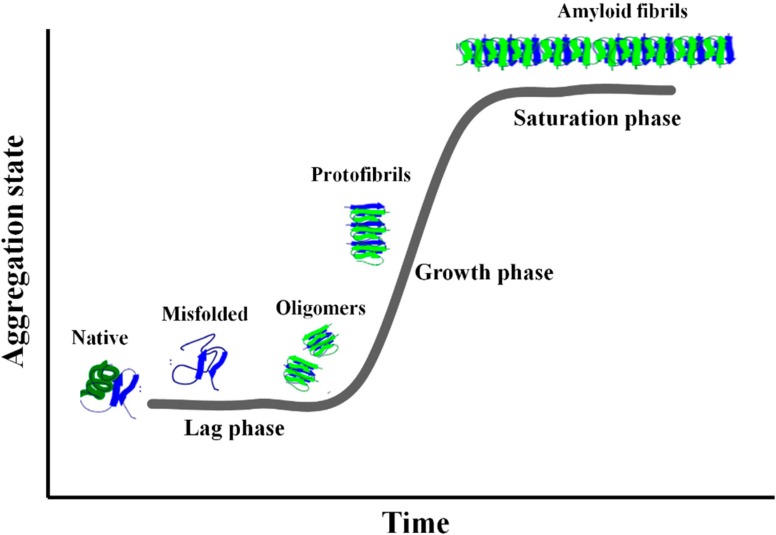
Nucleation-dependent fibril formation process The sequence of events along the fibril formation pathway includes: (Lag phase) aggregation of misfolded monomers into small intermediate oligomers; (Growth phase) re-arrangement of these oligomers into an organized conformation containing the cross beta structure; (Saturation phase) association of beta structured oligomers into proto-fibrils and finally into fibrils.

Amyloid diseases differ from each other in the specific protein deposited in the extracellular space and the specific tissues affected by protein deposition and degeneration [17]. Amyloid fibrils share common structural features despite the considerable diversity in the primary sequence of the constituent proteins. Amyloid deposits extracted from tissues are typically composed of unbranched fibrils (7 to 10 nm in diameter) assembled from two to three 3 nm filaments (protofilaments) twisted around each other. They are rich in β-sheet structures and the ordered regions adopt a cross-β structure in which individual strands in the β-sheets run perpendicular to the long axis of the fibril with the inter β-sheet hydrogen bonds oriented parallel to the fibril axis [18,19,20,21]. Amyloid fibril formation in bulk solution generally occurs through a nucleation-dependent polymerization process consisting of two phases, *i.e.*, nucleation (lag phase) and extension (growth phase) (Figure 1). The lag phase is assumed to be the time required for “nuclei” to form. The initial step of nucleus formation consists in the slow and reversible association of monomers.

This process is thermodynamically unfavorable and it is the rate limiting step of the fibrillation process. Once a nucleus has formed, the further addition of monomers to the nucleus becomes thermodynamically favored and this results in a rapid extension of amyloid fibrils [22]. Alternative amyloid growth pathways have been proposed, which are distinct from and compete with the nucleation-dependent pathway. In the nucleated conformational conversion model, it is supposed that spherical, fluid-like oligomeric complexes are rapidly formed and slowly convert into fibrils [23]. The oligomer assembly mediates a conformational transition of the amyloidogenic polypeptide from nearly random coil to β-sheet followed by the subsequent amyloid formation. This mechanism has been proposed for human amyloidogenic peptides such as islet amyloid polypeptide (IAPP) and Aβ peptide [24,25]. Another proposed mechanism is the monomer directed conversion in which the structural transition from a native state to a pre-fibrillar state undergone by a monomer influences other native state monomers to undergo the same transition, forming an intermediate amyloid fibrillar structure that then may grow into a fibril [26,27]. At present, it is hard to choice which of the proposed mechanisms is operating since few information on the intermediate structures present on the amyloid growth pathways is available [28,29].

Thus, the path of fibril formation begins with prefibrillar kinetic precursors, collectively indicated as protofibrils or soluble oligomeric intermediates, which appear as globules 2.5–5.0 nm in diameter or larger, with an intrinsic tendency to further organize into pore-like annular and tubular structures [30,31,32,33]. The interest for prefibrillar intermediates has recently grown, since, in most of cases, they have been associated to the higher cytotoxicity, whereas mature fibrils appeared less toxic or even harmless [30,34,35,36]. These results have led to the idea that the molecular basis of cell and tissue impairment may be related to the transient appearance of prefibrillar assemblies [37]. The specific mechanism by which these species appear to mediate their toxic effects is not completely understood; probably, toxicity is mediated by common structural features shared by prefibrillar precursors [28,29,30,31,32,33,34,35,36,37,38,39,40,41].

Each disease-specific amyloid contains an unique polypeptide that, through a complex and poorly understood mechanism, becomes misfolded *in vivo* forming prefibrillar aggregates that eventually assemble into highly ordered tissue deposits. Despite the implication of specific proteins in certain diseases, increasing evidence support the notion that all polypeptides have intrinsic properties that enable amyloid progression [42,43,44,45]. A recent genome-wide sequence survey identified the “amylome”, by which fibril-forming proteins constitutes roughly 15% of all coding polypeptides from *Escherichia coli* to humans [46].

## 3. GAGs and Amyloid Deposition

**Figure 2:**
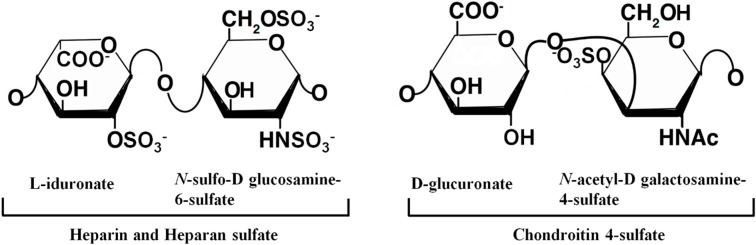
GAGs are highly negatively charged molecules, with extended conformation consisting of disaccharide repeating subunits. The disaccharide units of heparin contains *N*-acetylglucosamine (GlcNAc) and an uronic acid such as glucuronate or iduronate. Most glucosamine residues are bound in sulfamide linkage. Sulfate is also found on C-3 or C-6 of glucosamine and C-2 of uronic acid. HS contains the same disaccharide units as heparin except some glucosamine are acetylated and there are fewer sulfate groups. The disaccharide unit of CS contains *N*-acetylgalactosamine with sulfate on either C-4 or C-6 and glucuronate.

GAGs are the most abundant heteropolysaccharides in the human body. They are long, unbranched molecules consisting of disaccharide repeating subunits, having molecular weights of roughly 10–100 kDa. There are two main types of GAGs: non-sulfated GAGs that include hyaluronic acid, and sulfated GAGs that include chondroitin sulfate (CS), dermatan sulfate, keratan sulfate, heparin and heparan sulfate (HS). The main GAGs involved in amyloidosis are shown in Figure 2.

GAGs are highly negatively charged molecules, with an extended conformation that imparts high viscosity to the solution. GAGs are located primarily on the surface of cells or in the extracellular matrix of multicellular organisms, where they can be found either covalently linked to the protein core of proteoglycans or as free macromolecules. GAGs have been found to be closely associated with amyloid fibrils isolated from humans, and there is evidence that they play an active role in favoring amyloid fibril formation and stabilization [1,8,9]. Snow and Kisilevsky [47] reported an increase in GAGs levels at the time of serum amyloid A deposition. More recently, it has been demonstrated that inhibition of HS biosynthesis is directly related to loss of amyloid deposition in amyloid A animal models [48,49,50].

Evidence for the relation between GAGs and amyloid formation also comes from *in vitro* studies. GAGs, particularly HS and its highly sulfated derivative heparin, stimulate the formation of amyloid fibrils from the Alzheimer Aβ peptide *in vitro* [12,51,52]. In particular, GAGs interaction with Aβ1–40 and Aβ1–42 peptides was examined to identify their effect on peptide conformation and fibril formation. It was found that, in the presence of heparin, the random-coil to amyloidogenic β-sheet transition of Aβ peptide is accelerated, with Aβ1–42 rapidly adopting a β-sheet conformation. This acceleration was accompanied by the appearance of well-defined amyloid fibrils, indicating an enhanced nucleation of Aβ1–42. These findings clearly indicate that GAGs act at the earliest stage of fibril formation. Incubation of preformed Aβ1–42 fibrils with GAGs resulted in extensive lateral aggregation and deposition of the fibrils [53,54,55]. The sulfate moiety of GAGs is critical for Aβ fibril formation enhancement, its complete removal leading to a complete loss of promoting effect [51,56]. Low-molecular-weight heparins (LMWHs) can reverse the process of amyloidosis by inhibiting fibril formation and blocking the formation of β-plated structures, suggesting a possible role in a therapeutic approach interfering with the interaction between proteoglycans and Aβ peptides [57,58]. Scholefield *et al.* [59] reported that heparin is able to inhibit the activity of the protease responsible for β-secretase activity in neurons, *i.e.*, the β-site APP-cleaving enzyme 1 (BACE-1), whose activity is crucial to the amyloidogenic processing of APP resulting in the formation of the amyloidogenic Aβ peptide. Prefibrillar, oligomeric, soluble assemblies of Aβ are currently considered as toxic forms of the peptide. In particular, Aβ42 dimers and trimers appear to cause disruption of cognitive functions [60] and seem to exert their toxicity intracellularly [61,62]. Sandwall *et al.* [63] suggested a possible role of cell surface HS in mediating Aβ internalization and toxicity These Authors reported that HS-deficient cells were essentially resistant to Aβ toxicity and did not internalize the peptide; Aβ40 toxicity was also attenuated in cells over expressing heparanase. Moreover, addition of heparin to cells prevented internalization of added Aβ40, thus protecting against Aβ toxicity.

Heparin and, to a lesser extent, HS have been reported to increase significantly the rate of fibrillation also for tau protein [64,65], α-synuclein [66], gelsolin [67], β2-microglobulin [68,69] acyl-phosphatase [13], IAPP [70], immunoglobulin light-chain protein [71] and the aortic amyloid polypeptide medin [72]. De Carufel *et al.* [73] have recently observed that the interaction of heparin with IAPP causes a conformational transition from a random coil to an intermediate helical state which was postulated to be on the fibril formation pathway. Moreover, using cell line deficient in the biosynthesis of GAGs, it was observed that the lack of GAGs at the plasma membrane did not prevent IAPP-induced toxicity, whereas the presence of soluble heparin in the cell media inhibited IAPP cytotoxicity. The experimental evidence reported by these authors strongly corroborates the idea that sulfated GAGs accelerate the amyloid fibril formation process and let to postulate their active role in protecting cells against the cytotoxic prefibrillar oligomeric species formed at the beginning of the process. HS has also been found to convert the prion protein from the PrP^C^ to the PrP^SC^ form [74].More recently, it has been reported that GAGs exhibit a paradoxical effect, as they affect the aggregation rate of PrP, but also exert protective activity against prion conversion. In particular, it was found that low-molecular-weight heparin leads to transient PrP aggregation resulting in a soluble complex with a higher thermal stability compared to the native PrP. At the same time, increasing the PrP stability, GAGs are also able to exert a protective effect as these species are less susceptible to amyloid aggregation compared to the free protein [75].

Generally, among GAGs, heparin is particularly effective in accelerating fibril formation probably because of its high content of sulfate groups [51]. Several studies have demonstrated that electrostatic interactions are important in the binding of heparin to amyloid fibrils. In particular, removal of all sulfate groups from heparin or the addition of magnesium or calcium ions suppresses these interactions, thereby indicating their electrostatic nature [51,69]. Moreover, it has been postulated that the amyloid promoting activity of heparin is facilitated through specific amyloid polypeptide heparin interactions via binding sites [76,77,78,79,80,81]. Notwithstanding the large body of data associating heparin and other GAGs with amyloidogenesis, little is known about the mechanism by which heparin promotes amyloid formation or its effect on the overall aggregation pathway. Motamedi-Shad *et al.* [13] showed that heparan sulfate accelerates the conversion of acylphosphatase from the native state into the amyloidogenic, yet monomeric, partially folded state. The Authors also indicate that heparan sulfate does not simply accelerate the conversion of the resulting partially folded state into amyloid species, but splits the process into two distinct pathways occurring in parallel: a very fast phase in which heparan sulfate interacts with a fraction of protein molecules causing their rapid aggregation into β-sheet containing oligomers; and a slow phase resulting from the normal aggregation of partially folded molecules that cannot interact with heparan sulfate. Overall, the results indicate that heparan sulfate can both destabilize the initial folded state, accelerating its transition to the aggregation prone state, as well as cause a manifold acceleration on the subsequent self-assembly of partially unfolded monomers into amyloid oligomers.

**Figure 3:**
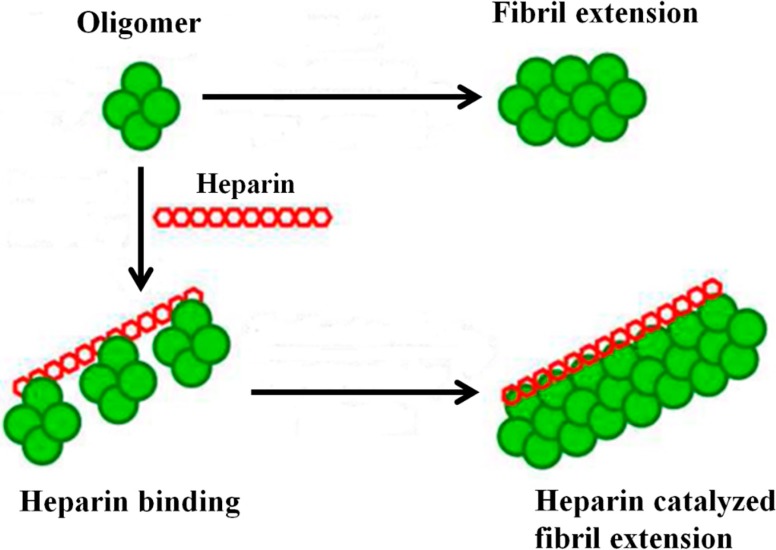
Alignment of oligomers on heparin molecules accelerates the process of fibril formation. Adapted from Solomon *et al.* [83].

More recently, Bourgault *et al.* [82] proposed that sulfated GAGs accelerate transthyretin (TTR) amyloidogenesis without influencing the initial steps of the TTR amyloidogenesis cascade, which includes tetramer dissociation, partial misfolding of the released monomer to form the amyloidogenic monomer, and formation of TTR oligomers. The sulfated polymeric surface of GAGs interacts with TTR oligomers, primarily through electrostatic interactions, concentrating TTR oligomers and possibly orienting them so as to accelerate the formation of larger aggregates by quaternary structural conversion (Figure 3).

The high density of sulfate groups and the polymeric nature of GAGs seem to be essential for binding to multiple TTR oligomers simultaneously and converting them into higher molecular weight aggregates, possibly by preferentially aligning them. The binding of heparin to amyloidogenic proteins has been reported to increase the degree of order of the protein within the aggregates, thus favoring the fibrillation process [69].

## 4. Molecular Recognition of Heparin by Proteins

Several studies have identified common structural features in the heparin/heparan sulfate binding sites in proteins. Different structural (NMR spectroscopy and X-ray crystallography) and molecular modeling approaches have been used to elucidate the GAG-protein interactions [84]. Side-chains of Asn, Asp, Glu, Gln, Arg, His and Trp are more likely to form the binding sites for non-sulfated carbohydrates than other amino acids [85,86,87]. The aromatic indole ring of Trp residue can pack against the hydrophobic face of a sugar molecule and has a significantly higher mean solvent accessibility in carbohydrate binding locations. Aliphatic residues side chains of Ala, Gly, Ile and Leu, which are usually buried inside proteins, do not appear to participate in sugar binding.

Clusters of positively charged amino acids can form ion pairs with spatially defined negatively charged sulfate or carboxylate groups on heparin chains. GAGs interact with residues that are prominently exposed on the surface of proteins. The main contribution to the binding affinity comes from ionic interactions between the highly acidic sulfate groups and the basic side chains of Arg, Lys and, to a lesser extent, His [88]. The relative strength of heparin binding by basic amino acid residues has been compared and arginine has been shown to bind 2.5 times more tightly than lysine. The guanidino group in arginine forms more stable hydrogen bonds as well as stronger electrostatic interactions with the sulfate groups. The ratio of these two residues determines, in part, the affinity of a binding site in a protein for GAGs [89].

Although the interactions between GAGs and proteins also involve different types of interactions, including van der Waals forces, hydrogen bonds and hydrophobic interactions with the carbohydrate backbone, the formation of ion pairs between positively charged side-chains and negatively charged GAG’s groups is certainly the most prominent cause of GAG-protein interaction. Also, it has been observed that heparin binding domains contain amino acids such as asparagine and glutamine which are capable of hydrogen bonding. The affinity of heparin-binding proteins for heparin/ HS is also enhanced because of the presence of polar residues with smaller side chains like serine and glycine.

Cardin *et al.* determined the structure of the heparin-binding regions in apolipoprotein B-100, the major protein constituent of human plasma low density lipoproteins (LDL) [90]. They showed that LDL contains five to seven heparin-binding sites of high positive charge density, the amino acid sequences of which were determined [91,92]. The same regions were also identified by Weisgraber and Rail [93]. The amino acid sequence of the heparin binding regions in apolipoprotein B-100 was found very similar to that of apolipoprotein E1819 and human vitronectin 20 with respect to the organization of basic and hydropathic residues [94,95]. Starting from these considerations, Cardin and Weintraub [96] suggested that heparin binding domains usually contain the consensus sequences XBBBXXBX or XBBXBX, where B is a lysine or arginine (with a very rare occurrence of His) and X is a non basic amino acid. The residues Asn, Ser, Ala, Gly, Ile, Leu and Tyr are more common at positions ‘X’. Residues such as Cys, Glu, Asp, Met, Phe and Trp exhibit a very low occurrence at positions ‘X’ in either α-helical or β-sheet domains of heparin binding proteins. Depending on the secondary structure of the protein, very few residues in these consensus sequences may actually participate in GAG binding sites. In particular, if the consensus sequence XBBBXXBX belong to an α-helix, basic side chains are usually displayed on one side forming an amphiphatic helical arrangement. Therefore, in order to interact with a linear GAG chain, it would be predicted that the positively charged amino acid residues in α-helical proteins would have to line up along the same side of the protein segment.

In β-strands, the positively charged residues in a GAG-binding protein are located in a different place compared with α-helical structures. The basic amino acids in the sequence XBBXBX line up on one face of a β-strand, whereas the hydropathic residues points back into the protein core. However, many proteins that bind heparin do not possess these sequences [95,97]. One model has suggested that a spacing of 20 Å between two basic amino acids is a critical determinant of heparin binding ability [98]. Such spacing can be achieved by peptide in α-helical conformation by basic amino acids spaced 13 residues apart or, in β-strand conformation, seven residues apart.

Clusters of basic amino acid residues capable of binding to the negatively charged heparin molecules have been also described in a variety of proteins that are induced to form β-structure upon heparin interaction. Recently, a novel structural signature for heparin-binding proteins has been proposed. The motif which has been named the CPC clip motif involves two cationic residues (Arg or Lys) and a polar residue (preferentially Asn, Gln, Thr, Tyr or Ser, more rarely Arg or Lys), with fairly conserved distances between the α-carbons and the side chain center of gravity, defining a clip-like structure where heparin would be lodged. This structural motif is highly conserved in a great number of heparin-binding protein structures available in the Protein Data Bank and can be found in many other proteins with reported heparin binding capacity [99]. The CPC clip motif has been found to correctly describe the heparin-binding sites of chemokines and human amyloid β protein.

## 5. Effect of GAGs on Peptides and Proteins Having a Weak or None Propensity to Aggregate

Several proteins non related to amyloid diseases have been reported to undergo amyloid fibril aggregation.

### 5.1. Apomyoglobin

Apomyoglobin is an eight alpha-helix protein that does not show any tendency to aggregate and to form fibrils under physiological conditions. It contains three consensus sequences corresponding to the consensus sequences identified by Cardin and Weintraub that are localized in the turn regions between helices C-D, E-F, and F-G. Moreover, clusters of basic amino acids that do not correspond to consensus sequences are present in the primary structure at the end of the B helix, *i.e.*, RLFKSH, the beginning of the E helix, *i.e.*, LKKHG, and at the end of the G-helix, *i.e.*, HVLHSRH. ThT reactive aggregates are readily formed following the addition of heparin to apomyoglobin indicating amyloid aggregation and fibril formation that is much more evident on lowering the pH from 7.0 to 5.5 [100]. The effect of the heparin-induced wild-type apomyoglobin aggregates on cell viability was also examined. Protein aggregates formed at the beginning of the aggregation process at pH 7.0 were able to kill about 60% of cultured cells, whereas aggregates formed at pH 5.5 were harmless. Six days after aggregation onset, the aggregates formed at both pH values were not cytotoxic. The different cytotoxicity of the aggregates formed at the beginning of the aggregation process at pH 7.0 and pH 5.5 could be related to their different compactness. Indeed, at pH 7.0 the low number of electrostatic interactions between heparin and protonated hystidyl residues makes the aggregates less compact thereby determining an increase of their exposed hydrophobic area [101]. This different toxicity could also be due to an acceleration of the fibrillization process that occurs at pH 5.5. In fact, it is highly likely that, under these conditions, a reduced steady-state level of early toxic aggregates is reached consequently to the increased rate of oligomer growth into harmless higher order assemblies, as recently reported for TTR aggregation in the presence of heparin [82]. The observation that addition of salt at pH 5.5 does not influence the heparin induced aggregation profile indicates that the increased aggregation is not only related to the histidine protonation but also to a greater propensity of the protein to undergo structural modifications. It has been shown that lowering pH from 7.0 to 5.5 reduces the conformational stability of apomyoglobin of around 2–3 kcal/mole thereby making the protein more susceptible to perturbing agents [102]. In this context, the proton gradient formed in proximity of the heparin surface is likely to modify the structural properties of the protein and possibly favor its misfolding. Taken together, the results obtained with wild-type apomyoglobin indicate that heparin is able to induce an amyloid aggregation process that readily ends with the formation of a fibrillar species rich in cross-β structure [100].

### 5.2. 23-Residue Peptide of the Phospholamban Transmembrane Protein (PLB(1–23))

Recently, Madine and coworkers [103] investigated the effect of heparin as a cofactor to induce amyloid-like fibril formation in a natively unfolded peptide, *i.e.*, the 23-residue peptide PLB(1–23). This peptide, corresponding to the acetylated cytoplasmic domain of the phospholamban trans-membrane protein, is predicted to have a weak propensity to aggregate and it is not associated with amyloid disorders. In the presence of low-molecular mass (5 kDa) heparin, the peptide undergoes spontaneous and rapid assembly into amyloid-like fibrils, this effect is more pronounced at pH 5.5 than at pH 7.4. At lower pH values, peptide aggregation is associated to a transition in a β-rich structure. These results are consistent with the hypothesis that polyanionic heparin works as scaffold in enhancing aggregation by aligning the peptide molecules in the correct orientation and with the appropriate periodicity. PLB(1–23), in its soluble form, is toxic to cells and the promotion of fibril formation by heparin reduces the toxicity of this peptide, consistent with the notion that amyloid-like fibrils represent an harmless end stage of fibrillization.

### 5.3. Islet Amyloid Polypeptide (IAPP) Variants

Islet amyloid polypeptide (IAPP) is one of the most amyloidogenic, naturally occurring polypeptides and amyloid formation is accelerated in the presence of model membranes containing anionic lipids. Heparin and, to a lesser extent, HS have been reported to increase significantly the rate of fibrillation of IAPP [70], but very little is known about GAGs ability in inducing amyloid formation in apparently IAPP non-amyloidogenic variants. Recently, Wang and coworkers [104] reported that IAPP non-amyloidogenic variants form amyloid in the presence of GAGs, this effect being more effective than that of anionic lipid vesicles. In particular, the I26P mutant of human IAPP (I26P-IAPP) renders the protein non-amyloidogenic and converts it into an inhibitor of amyloid formation for the wild-type IAPP [105]. Similarly, the doubly *N*-methylated variant of human IAPP, G24-*N*-methyl, I26-*N*-methyl-IAPP (NMe-G24, NMe-I26-IAPP), behaves as a potent inhibitor of IAPP amyloid formation in homogeneous solution and it is itself non amyloidogenic [106]. The molecular basis of the inhibitory effect are still unknown, but all mutations are located in a region that has been shown to be responsible for amyloid formation [107,108]. Several known inhibitors of IAPP amyloid formation have been shown to be less effective in the presence of GAGs. These data further confirm the role that heparin and other GAGs plays in enhancing amyloid formation in a range of amyloid-related proteins and provide therapeutic strategies aiming at the reduction of cytotoxicity of amyloidogenic species along the amyloid formation pathway.

### 5.4. N-Terminal Domain of Escherichia coli HypF (HypF-N)

HypF-N is a 91-residue peptide, with no disulfide bridges or cofactors and apparently unrelated to any protein deposition disease [109]. Nevertheless, under *in vitro* conditions that destabilize its native globular form, such as low pH or presence of small to moderate concentrations of trifluoroethanol (TFE), HypF-N can aggregate into amyloid-like fibrils through the formation of toxic prefibrillar oligomers [110]. Recently, Saridaki and coworkers [111] reported that GAGs were not able to bind preformed HypF-N oligomers, to change their morphological and structural characteristics or to disaggregate them. Notably, GAGs were found to bind the cell surface preventing the interaction between the oligomers and the cells. These studies suggest that suppression of the oligomer toxicity by GAGs may involve different mechanisms such as direct binding to the oligomers with a consequent acceleration of harmful fibril formation or through mechanisms independent of direct GAG-oligomers binding as observed for HypF-N aggregates.

## 6. Conclusions and Perspectives

A number of challenges lie ahead in the investigation of the interactions between GAGs and proteins from a structural and functional point of view. For example, heparin is known to interact with a number of proteins but the precise mechanism of interaction and the induced effect are not clearly understood. Acceleration of fibril formation seems to reduce the cytotoxicity associated with the intermediate species formed along the fibril formation pathway [21,72,112,113]. In this respect, the enhancement of fibril formation induced by GAGs with a consequent reduction of highly cytotoxic species may thus provide a therapeutic strategy for targeting amyloid-related diseases. Moreover, the recent observation that heparin does not directly bind or alter the structural and morphological properties of preformed protein oligomers suggests that exogenous heparin may interact with the cell surface preventing the interaction of these aggregates with the cell membrane [111]. However, Holmes *et al.* [114] have recently reported that heparan sulfate proteoglycans (HSPGs) mediate internalization and propagation of specific proteopathic seeds formed by α-synuclein and tau. The mechanism involves the release of protein aggregates into the extracellular space which then enter neighboring cells via HSPG binding to seed further fibrillization.

The identification of the structure-activity relationships between amyloidogenic sequences and different GAGs as well as the conditions under which GAG binding occurs, are also needed for developing specific GAG therapeutic interventions [57,115,116]. In particular, soluble proteins, which normally do not show any tendency to aggregate can be induced to form amyloid aggregates by GAGs. Thus, GAGs could play a dual role in amyloidosis, as a safe compound, inducing the protein to assume a non-toxic fibrillar conformation, and as a pathological chaperone, inducing protein aggregation. Another aspect is whether the fibrillization-accelerating effect of GAGs can be effectively used as a therapeutic target in patients affected by amyloid-associated diseases or if they are deleterious for health because of the increase of fibril load.

## References

[1] Van Horssen J., Wesseling P., van den Heuvel L.P., de Waal R.M., Verbeek M.M. (2003). Heparan sulphate proteoglycans in Alzheimer’s disease and amyloid-related disorders. Lancet Neurol..

[2] Snow A.D., Wight T.N. (1989). Proteoglycans in the pathogenesis of Alzheimer’s disease and other amyloidosis. Neurobiol. Aging.

[3] Inoue S. (2001). Basement membrane and beta amyloid fibrillogenesis in Alzheimer’s disease. International Review of Cytology—A Survey of Cell Biology.

[4] Potter-Perigo S., Hull R.L., Tsoi C., Braun K.R., Andrikopoulos S., Teague J., Verchereb C.B., Kahnb S.E., Wight T.N. (2003). Proteoglycans synthesized and secreted by pancreatic islet beta-cells bind amylin. Arch. Biochem. Biophys..

[5] Ancsin J.B. (2003). Amyloidogenesis: Historical and modern observations point to heparan sulfate proteoglycans as a major culprit. Amyloid.

[6] Young I.D., Ailles L., Narindrasorasak S., Tan R., Kisilevsky R. (1992). Localization of the basement membrane heparan sulfate proteoglycan in islet amyloid deposits in type II diabetes mellitus. Arch. Pathol. Lab. Med..

[7] Snow A.D., Wight T.N., Nochlin D., Koike Y., Kimata K., de Armond S.J., Prusiner S.B. (1990). Immunolocalization of heparan sulfate proteoglycans to the prion protein amyloid plaques of Gerstmann-Straussler syndrome, Creutzfeldt-Jakob disease and scrapie. Lab. Investig..

[8] Diaz-Nido J., Wandossel F., Avila J. (2002). Glycosaminoglycans and beta-amyloid, prion and tau peptides in neurodegenerative diseases. Peptides.

[9] Gruys E., Ultee A., Upragarin N. (2006). Glycosaminoglycans are part of amyloid fibrils: Ultrastructural evidence in avian AA amyloid stained with cuprolinic blue and labeled with immunogold. Amyloid.

[10] Papy-Garcia D., Christophe M., Huynh M.B., Fernando S., Ludmilla S., Sepulveda-Diaz J.E., Raisman-Vozari R. (2011). Glycosaminoglycans, protein aggregation and neurodegeneration. Curr. Protein Pept. Sci..

[11] Zhu M., Souillac P.O., Ionesco-Zanetti C., Carter S.A., Fink A.L. (2002). Surface-catalyzed amyloid fibril formation. J. Biol. Chem..

[12] McLaurin J., Franklin T., Zhang X., Deng J., Fraser P.E. (1999). Interactions of Alzheimer amyloid-beta peptides with glycosaminoglycans effects on fibril nucleation and growth. Eur. J. Biochem..

[13] Motamedi-Shad N., Monsellier E., Chiti F. (2009). Amyloid formation by the model protein muscle acylphosphatase is accelerated by heparin and heparan sulphate through a scaffolding-based mechanism. J. Biochem..

[14] Taylor J.P., Hardy J., Fischbeek K.H. (2005). Toxic proteins in neurodegenerative disease. Science.

[15] Chiti F., Dobson C. (2006). Protein misfolding, functional amyloid, and human diseases. Annu. Rev. Biochem..

[16] Sipe J.D., Benson M.D., Buxbaum J.N., Ikeda S., Merlini G., Saraiva M.J., Westermark P. (2010). Amyloid fibril protein nomenclature: 2010 Recommendations of the nomenclature committee of the international society of amyloidosis. Amyloid.

[17] Sekijima Y., Wiseman R.L., Matteson J., Hammarström P., Miller S.R., Sawkar A.R., Balch W.E., Kelly J.W. (2005). The biological and chemical basis for tissue-selective amyloid disease. Cell.

[18] Sunde M., Blake C. (1997). The structure of amyloid fibrils by electron microscopy and X-ray diffraction. Adv. Protein Chem..

[19] Makin O.S., Serpell L.C. (2002). Examining the structure of the mature amyloid fibril. Biochem. Soc. Trans..

[20] Nelson R., Sawaya M.R., Balbirnie M., Madsen A.O., Riekel C., Grothe R., Eisenberg D. (2005). Structure of the cross-beta spine of amyloid-like fibrils. Nature.

[21] Iannuzzi C., Maritato R., Irace G., Sirangelo I. (2013). Misfolding and amyloid aggregation of apomyoglobin. Int. J. Mol. Sci..

[22] Kelly J.W. (1998). The alternative conformations of amyloidogenic proteins and their multi-step assembly pathways. Curr. Opin. Struct. Biol..

[23] Serio T.R., Cashikar A.G., Kowal A.S., Sawicki G.J., Moslehi J.J., Serpell L., Arnsdorf M.F., Lindquist S.L. (2000). Nucleated conformational conversion and the replication of conformational information by a prion determinant. Science.

[24] Wei L., Jiang P., Xu W., Li H., Zhang H., Yan L., Chan-Park M.B., Liu X.W., Tang K., Mu Y. (2011). The molecular basis of distinct aggregation pathways of islet amyloid polypeptide. J. Biol. Chem..

[25] Lee J., Culyba E.K., Powers E.T., Kelly J.W. (2012). Amyloid-β forms fibrils by nucleated conformational conversion of oligomers. Nat. Chem. Biol..

[26] Caughey B., Lansbury P.T. (2003). Protofibrils, pores, fibrils, and neurodegeneration: Separating the responsible protein aggregates from the innocent bystanders. Ann. Rev. Neurosci..

[27] Gibson T.J., Murphy R.M. (2006). Inhibition of insulin fibrillogenesis with targeted peptides. Prot. Sci..

[28] Bernacki J.P., Murphy R.M. (2009). Model discrimination and mechanistic interpretation of kinetic data in protein aggregation studies. Biophys. J..

[29] Phelps E.M., Hall C.K. (2012). Structural transitions and oligomerization along polyalanine fibril formation pathways from computer simulations. Proteins.

[30] Lashuel H.A., Petre B.M., Wall J., Simon M., Nowark R.J., Waltz T., Lansbury P.T. (2002). Alpha-synuclein, especially the Parkinson’s disease-associated mutants, forms pore-like annular and tubular protofibrils. J. Mol. Biol..

[31] Poirier M.A., Li H., Macosko J., Cail S., Amzel M., Ross C.A. (2002). Huntingtin spheroids and protofibrils as precursors in polyglutamine fibrilization. J. Biol. Chem..

[32] Cohen S.I., Vendruscolo M., Dobson C.M., Knowles T.P. (2012). From macroscopic measurements to microscopic mechanisms of protein aggregation. J. Mol. Biol..

[33] Ortore M.G., Spinozzi F., Vilasi S., Sirangelo I., Irace G., Shukla A., Narayanan T., Sinibaldi R., Mariani P. (2011). Time-resolved small-angle X-ray scattering study of the early stage of amyloid formation of an apomyoglobin mutant. Phys. Rev. E Stat. Nonlinear Soft Matter Phys..

[34] Lambert M.P., Barlow A.K., Chromy B.A., Edwards C., Freed R., Liosatos M., Morgan T.E., Rozovsky I., Trommer B., Viola K.L. (1998). Diffusible, non fibrillar ligands derived from Abeta1–42 are potent central nervous system neurotoxins. Proc. Natl. Acad. Sci. USA.

[35] Walsh D.M., Selkoe D.J. (2000). Oligomers on the brain: The emerging role of soluble protein aggregates in neurodegeneration. Protein Pept. Lett..

[36] Reixach N., Deechongkit S., Jiang X., Kelly J.W., Buxbaum J.N. (2004). Tissue damage in the amyloidosis: Transthyretin monomers and nonnative oligomers are the major cytotoxic species in tissue culture. Proc. Natl. Acad. Sci. USA.

[37] Cecchi C., Stefani M. (2013). The amyloid-cell membrane system. The interplay between the biophysical features of oligomers/fibrils and cell membrane defines amyloid toxicity. Biophys. Chem..

[38] Malmo C., Vilasi S., Iannuzzi C., Tacchi S., Cametti C., Irace G., Sirangelo I. (2006). Tetracycline inhibits W7FW14F apomyoglobin fibril extension and keeps the amyloid protein in a pre-fibrillar, highly cytotoxic state. FASEB J..

[39] Bucciantini M., Calloni G., Chiti F., Formigli L., Nosi D., Dobson C.M., Stefani M. (2004). Prefibrillar amyloid protein aggregates share common features of cytotoxicity. J. Biol. Chem..

[40] Kayed R., Head E., Thomson J.L., Mcintire T.M., Milton S.C., Cotman C.W., Glabe C.G. (2003). Common structure of soluble amyloid oligomers implies common mechanism of pathogenesis. Science.

[41] Stefani M. (2010). Biochemical and biophysical features of both oligomer/fibril and cell membrane in amyloid cytotoxicity. FEBS J..

[42] Guijarro J.I., Sunde M., Jones J.A., Campbell I.D., Dobson C.M. (1998). Amyloid fibril formation by an SH3 domain. Proc. Natl. Acad. Sci. USA.

[43] Litvinovich S.V., Brew S.A., Aota S., Akiyama S.K., Haudenschild C., Ingham K.C. (1998). Formation of amyloid-like fibrils by self-association of a partially unfolded fibronectin type III module. J. Mol. Biol..

[44] Fandrich M., Fletcher M.A., Dobson C.M. (2001). Amyloid fibrils from muscle myoglobin. Nature.

[45] Infusini G., Iannuzzi C., Vilasi S., Birolo L., Pagnozzi D., Pucci P., Irace G., Sirangelo I. (2012). Resolution of the effects induced by W→F substitutions on the conformation and dynamics of the amyloid-forming apomyoglobin mutant W7FW14F. Eur. Biophys. J..

[46] Goldschmidt L., Teng P.K., Riek R., Eisenberg D. (2010). Identifying the amylome, proteins capable of forming amyloid-like fibrils. Proc. Natl. Acad. Sci. USA.

[47] Snow A.D., Kisilevsky R. (1985). Temporal relationship between glycosaminoglycan accumulation and amyloid deposition during experimental amyloidosis. A histochemical study. Lab. Investig..

[48] Kisilevsky R., Szarek W.A., Ancsin J.B., Elimova E., Marone S., Bhat S., Berkin A. (2004). Inhibition of amyloid A amyloidogenesis *in vivo* and in tissue culture by 4-deoxy analogues of peracetylated 2-acetamido-2-deoxy-alpha- and beta-d-glucose: Implications for the treatment of various amyloidosis. Am. J. Pathol..

[49] Elimova E., Kisilevsky R., Szarek W.A., Ancsin J.B. (2004). Amyloidogenesis recapitulated in cell culture: A peptide inhibitor provides direct evidence for the role of heparan sulfate and suggests a new treatment strategy. FASEB J..

[50] Li J.P., Galvis M.L., Gong F., Zhang X., Zcharia E., Metzger S., Vlodavsky I., Kisilevsky R., Lindahl U. (2005). *In vivo* fragmentation of heparan sulfate by heparanase overexpression renders mice resistant to amyloid protein A amyloidosis. Proc. Natl. Acad. Sci. USA.

[51] Castillo G.M., Lukito W., Wight T.N., Snow A.D. (1999). The sulfate moieties of glycosaminoglycans are critical for the enhancement of beta-amyloid protein fibril formation. J. Neurochem..

[52] Castillo G.M., Ngo C., Cummings J., Wight T.N., Snow A.D. (1997). Perlecan binds to the beta-amyloid proteins (A beta) of Alzheimer’s disease, accelerates A beta fibril formation, and maintains A beta fibril stability. J. Neurochem..

[53] Fraser P.E., Nguyen J.T., Chin D.T., Kirschner D.A. (1992). Effects of sulfate ions on Alzheimer beta/A4 peptide assemblies: Implications for amyloid fibril-proteoglycan interactions. J. Neurochem..

[54] Fraser P.E., Darabie A.A., McLaurin J.A. (2001). Amyloid-beta interactions with chondroitin sulfate-derived monosaccharides and disaccharides. Implications for drug development. J. Biol. Chem..

[55] McLaurin J., Fraser P.E. (2000). Effect of amino-acid substitutions on Alzheimer’s amyloid-beta peptide-glycosaminoglycan interactions. Eur. J. Biochem..

[56] Valle-Delgado J.J., Alfonso-Prieto M., de Groot N.S., Ventura S., Samitier J., Rovira C., Fernàndez-Busquets X. (2010). Modulation of Abeta42 fibrillogenesis by glycosaminoglycan structure. FASEB J..

[57] Ariga T., Miyatake T., Yu R.K. (2010). Role of proteoglycans and glycosaminoglycans in the pathogenesis of Alzheimer’s disease and related disorders: Amyloidogenesis and therapeutic strategies—A review. J. Neurosci. Res..

[58] Bergamaschini L., Rossi E., Vergani C., de Simoni M.G. (2009). Alzheimer’s disease: Another target for heparin therapy. Sci. World J..

[59] Scholefield Z., Yates E.A., Wayne G., Amour A., McDowell W., Turnbull J.E. (2003). Heparan sulfate regulates amyloid precursor protein processing by BACE1, the Alzheimer’s beta-secretase. J. Cell Biol..

[60] Walsh D.M., Klyubin I., Shankar G.M., Townsend M., Fadeeva J.V., Betts V., Podlisny M.B., Cleary J.P., Ashe K.H., Rowan M.J. (2005). The role of cell-derived oligomers of Abeta in Alzheimer’s disease and avenues for therapeutic intervention. Biochem. Soc. Trans..

[61] Gouras G.K., Tsai J., Naslund J., Vincent B., Edgar M., Checler F., Greenfield J.P., Haroutunian V., Buxbaum J.D., Xu H. (2000). Intraneuronal Abeta42 accumulation in human brain. Am. J. Pathol..

[62] Wirths O., Multhaup G., Bayer T.A. (2004). A modified beta-amyloid hypothesis: Intraneuronal accumulation of the beta-amyloid peptide-the first step of a fatal cascade. J. Neurochem..

[63] Sandwall E., O’Callaghan P., Zhang X., Lindahl U., Lannfelt L., Li J.P. (2010). Heparan sulfate mediates amyloid-beta internalization and cytotoxicity. Glycobiology.

[64] Goedert M., Jakes R., Spillantini M.G., Hasegawa M., Smith M.J., Crowther R.A. (1996). Assembly of microtubule-associated protein tau into Alzheimer-like filaments induced by sulphated glycosaminoglycans. Nature.

[65] Paudel H.K., Li W. (1999). Heparin-induced conformational change in microtubule-associated protein Tau as detected by chemical cross-linking and phosphopeptide mapping. J. Biol. Chem..

[66] Cohlberg J.A., Li J., Uverskky V.N., Fink A.L. (2002). Heparin and other glycosaminoglycans stimulate the formation of amyloid fibrils from alpha synuclein *in vitro*. Biochemistry.

[67] Suk J.Y., Zhang F., Balch W.E., Linhardt R.J., Kelly J.F. (2006). Heparin accelerates gelsolin amyloidogenesis. Biochemistry.

[68] Relini A., de Stefano S., Torrassa S., Cavalleri O., Rolandi R., Gliozzi A., Giorgetti S., Raimondi S., Marchese L., Verga L. (2008). Heparin strongly enhances the formation of beta2-microglobulin amyloid fibrils in the presence of type I collagen. J. Biol. Chem..

[69] Calamai M., Kumita J.R., Mifsud J., Parrini C., Ramazzotti M., Ramponi G., Taddei N., Chiti F., Dobson C.M. (2006). Nature and significance of the interactions between amyloid fibrils and biological polyelectrolytes. Biochemistry.

[70] Meng F., Abedini A., Song B., Raleigh D.P. (2007). Amyloid formation by pro-islet amyloid polypeptide processing intermediates: Examination of the role of protein heparan sulfate interactions and implications for islet amyloid formation in type 2 diabetes. Biochemistry.

[71] McLaughlin R.W., de Stigter J.K., Sikkink L.A., Baden E.M., Ramirez-Alvarado M. (2006). The effects of sodium sulfate, glycosaminoglycans, and Congo red on the structure, stability, and amyloid formation of an immunoglobulin light-chain protein. Protein. Sci..

[72] Madine J., Middleton D.A. (2010). Comparison of aggregation enhancement and inhibition as strategies for reducing the cytotoxicity of the aortic amyloid polypeptide medin. Eur. Biophys. J..

[73] De Carufel C.A., Nguyen P.T., Sahnouni S., Bourgault S. (2013). New insights into the roles of sulfated glycosaminoglycans in islet amyloid polypeptide amyloidogenesis and cytotoxicity. Biopolymers.

[74] Wong C., Xiong L.W., Horiuchi M., Raymond L., Wehrly K., Chesebro B., Caughey B. (2001). Sulfated glycans and elevated temperature stimulate PrP(Sc)-dependent cell-free formation of protease-resistant prion protein. EMBO J..

[75] Vieira T.C., Cordeiro Y., Caughey B., Silva J.L. (2014). Heparin binding confers prion stability and impairs its aggregation. FASEB J..

[76] Narindrasorasak S., Lowery D., Gonzalez-DeWhitt P., Poorman R.A., Greenberg B., Kisilevsky R. (1991). High affinity interactions between the Alzheimer’s beta-amyloid precursor proteins and the basement membrane form of heparan sulfate proteoglycan. J. Biol. Chem..

[77] Brunden K.R., Richter-Cook N.J., Chaturvedi N., Frederickson R.C. (1993). pH-Dependent binding of synthetic beta-amyloid peptides to glycosaminoglycans. J. Neurochem..

[78] Caughey B., Brown K., Raymond G.J., Katzenstein G.E., Thresher W. (1994). Binding of the protease-sensitive form of PrP (prion protein) to sulfated glycosaminoglycan and Congo red. J. Virol..

[79] Warner R.G., Hundt C., Weiss S., Turnbull J.E. (2002). Identification of the heparan sulfate binding sites in the cellular prion protein. J. Biol. Chem..

[80] Park K., Verchere C.B. (2001). Identification of a heparin binding domain in the N-terminal cleavage site of pro-islet amyloid polypeptide. Implications for islet amyloid formation. J. Biol. Chem..

[81] Ohashi K., Kisilevsky R., Yanagishita M. (2002). Affinity binding of glycosaminoglycans with beta(2)-microglobulin. Nephron.

[82] Bourgault S., Solomon J.P., Reixach N., Kelly J.W. (2011). Sulfated glycosaminoglycans accelerate transthyretin amyloidogenesis by quaternary structural conversion. Biochemistry.

[83] Solomon J.P., Bourgault S., Powers E.T., Kelly J.W. (2011). Heparin binds 8 kDa gelsolin cross-β-sheet oligomers and accelerates amyloidogenesis by hastening fibril extension. Biochemistry.

[84] Sasisekharan R., Raman R., Prabhakar V. (2006). Glycomis approach to structure-function relationships of glycosaminoglycans. Annu. Rev. Biomed. Eng..

[85] Malik A., Ahmad S. (2007). Sequence and structural features of carbohydrate binding in proteins and assessment of predictability using a neural network. BMC Struct. Biol..

[86] Shionyu-Mitsuyama C., Shirai T., Ishida H., Yamane T. (2003). An empirical approach for structure-based prediction of carbohydrate-binding sites on proteins. Protein. Eng. Des. Sel..

[87] Taroni C., Jones S., Thornton J.M. (2000). Analysis and prediction of carbohydrate binding sites. Protein. Eng. Des. Sel..

[88] Fromm J.R., Hileman R.E., Caldwell E.E.O., Weiler J.M., Linhardt R.J. (1997). Pattern and spacing of basic amino acids in heparin binding sites. Arch. Biochem. Biophys..

[89] Hileman R.E., Fromm J.R., Weiler J.M., Linhardt R.J. (1998). Glycosaminoglycan-protein interactions: Definition of consensus sites in glycosaminoglycan binding proteins. BioEssays.

[90] Cardin A.D., Randall C.J., Hirose N., Jackson R.L. (1987). Physical-chemical interaction of heparin and human plasma low-density lipoproteins. Biochemistry.

[91] Hirose N., Blankenship D.T., Krivanek M.A., Jackson R.L., Cardin A.D. (1987). Isolation and characterization of four heparin-binding cyanogen bromide peptides of human plasma apolipoprotein B. Biochemistry.

[92] Gigli M., Consonni A., Ghiselli G., Rizzo V., Naggi A., Torri G. (1992). Heparin binding to human plasma low-density lipoproteins: Dependence on heparin sulfation degree and chain length. Biochemistry.

[93] Weisgraber K.H., Rail S.C. (1987). Human apolipoprotein B-100 heparin-binding sites. J. Biol. Chem..

[94] Cardin A.D., Hirose N., Blankenship D.T., Jackson R.L., Harmony J.A., Sparrow D.A., Sparrow J.T. (1986). Binding of a high reactive heparin to human apolipoprotein E: Identification of two heparin-binding domains. Biochem. Biophys. Res. Commun..

[95] Weisgraber K.H., Rail S.C., Mahley R.W., Milne R.W., Marcel Y.L., Sparrow J.T. (1986). Human apolipoprotein E. Determination of the heparin binding sites of apolipoprotein E3. J. Biol. Chem..

[96] Cardin A.D., Weintraub H.J. (1989). Molecular modeling of protein-glycosaminoglycan interactions. Arteriosclerosis.

[97] Baird A., Schubert D., Ling N., Guillemin R. (1988). Three-dimensional structure of human basic fibroblast growth factor. Proc. Natl. Acad. Sci. USA.

[98] Margalit H., Fischer N., Ben-Sasson S.A. (1993). Comparative analysis of structurally defined heparin binding sequences reveals a distinct spatial distribution of basic residues. J. Biol. Chem..

[99] Torrent M., Nogués M.V., Andreu D., Boix E. (2012). The “CPC Clip Motif”: A conserved structural signature for heparin-binding proteins. PLoS One.

[100] Vilasi S., Sarcina R., Maritato R., de Simone A., Irace G., Sirangelo I. (2011). Heparin induces harmless fibril formation in amyloidogenic W7FW14F apomyoglobin and amyloid aggregation in wild-type protein *in vitro*. PLoS One.

[101] Campioni S., Mannini B., Pensalfini A., Zampagni M., Parrini C., Evangelisti E., Relini A., Stefani M., Dobson C.M., Cecchi C. (2010). A causative link between the structure of aberrant protein oligomers and their toxicity. Nat. Chem. Biol..

[102] Bismuto E., Colonna G., Irace G. (1983). Unfolding pathway of myoglobin. Evidence for a multistate process. Biochemistry.

[103] Madine J., Davies H.A., Hughes E., Middleton D.A. (2013). Heparin promotes the rapid fibrillization of a peptide with low intrinsic amyloidogenicity. Biochemistry.

[104] Wang H., Cao P., Raleigh D.P. (2013). Amyloid formation in heterogeneous environments: Islet amyloid polypeptide glycosaminoglycan interactions. J. Mol. Biol..

[105] Abedini A., Meng F.L., Raleigh D.P. (2007). A single-point mutation converts the highly amyloidogenic human islet amyloid polypeptide into a potent fibrillization inhibitor. J. Am. Chem. Soc..

[106] Yan L.M., Tatarek-Nossol M., Velkova A., Kazantzis A., Kapurniotu A. (2006). Design of a mimic of non amyloidogenic and bioactive human islet amyloid polypeptide (IAPP) as nanomolar affinity inhibitor of IAPP cytotoxic fibrillogenesis. Proc. Natl. Acad. Sci. USA.

[107] Westermark P., Engstrom U., Johnson K.H., Westermark G.T., Betsholtz C. (1990). Islet amyloid polypeptide: Pinpointing amino acid residues linked to amyloid fibril formation. Proc. Natl. Acad. Sci. USA.

[108] Shim S.H., Gupta R., Ling Y.L., Strasfeld D.B., Raleigh D.P., Zanni M.T. (2009). Two-dimensional IR spectroscopy and isotope labeling defines the pathway of amyloid formation with residue-specific resolution. Proc. Natl. Acad. Sci. USA.

[109] Rosano C., Zuccotti S., Bucciantini M., Stefani M., Ramponi G., Bolognesi M. (2002). Crystal structure and anion binding in the prokaryotic hydrogenase maturation factor HypF acylphosphatase-like domain. J. Mol. Biol..

[110] Chiti F., Bucciantini M., Capanni C., Taddei N., Dobson C.M., Stefani M. (2001). Solution conditions can promote formation of either amyloid protofilaments or mature fibrils from the HypF N-terminal domain. Protein Sci..

[111] Saridaki T., Zampagni M., Mannini B., Evangelisti E., Taddei N., Cecchi C., Chiti F. (2012). Glycosaminoglycans (GAGs) suppress the toxicity of HypF-N prefibrillar aggregates. J. Mol. Biol..

[112] Sirangelo I., Irace G. (2010). Inhibition of aggregate formation as therapeutic target in protein misfolding diseases: Effect of tetracycline and trehalose. Expert Opin. Ther. Targets.

[113] Iannuzzi C., Maritato R., Irace G., Sirangelo I. (2013). Glycation accelerates fibrillization of the amyloidogenic W7FW14F apomyoglobin. PLoS One.

[114] Holmes B.B., DeVos S.L., Kfoury N., Li M., Jacks R., Yanamandra K., Ouidja M.O., Brodsky F.M., Marasa J., Bagchi D.P. (2013). Heparan sulfate proteoglycans mediate internalization and propagation of specific proteopathic seeds. Proc. Natl. Acad. Sci. USA.

[115] Hirschfield G.M., Hawkins P.N. (2003). Amyloidosis: New strategies for treatment. Int. J. Biochem. Cell Biol..

[116] Zhang X., Li J.P. (2010). Heparan sulfate proteoglycans in amyloidosis. Prog. Mol. Biol. Transl. Sci..

